# Single-Droplet Microsensor for Ultra-Short Circulating EFGR Mutation Detection in Lung Cancer Based on Multiplex EFIRM Liquid Biopsy

**DOI:** 10.3390/ijms241210387

**Published:** 2023-06-20

**Authors:** Fang Wei, Peter Yu, Jordan Cheng, Feng Li, David Chia, David T. W. Wong

**Affiliations:** 1 School of Dentistry, University of California, Los Angeles, CA 90095, USA; jcheng1@ucla.edu (J.C.); fengli@ucla.edu (F.L.); dtww@ucla.edu (D.T.W.W.); 2 Department of Physics, University of California, Los Angeles, CA 90095, USA; peter.yu@physics.ucla.edu; 3 Department of Pathology, David Geffen School of Medicine, University of California, Los Angeles, CA 90095, USA

**Keywords:** biosensor, ultra-short circulating tumor DNA, lung cancer, liquid biopsy, EGFR

## Abstract

Liquid biopsy is a rapidly emerging field that involves the minimal/non-invasive assessment of signature somatic mutations through the analysis of circulating tumor DNA (ctDNA) shed by tumor cells in bodily fluids. Broadly speaking, the unmet need in liquid biopsy lung cancer detection is the lack of a multiplex platform that can detect a mutation panel of lung cancer genes using a minimum amount of sample, especially for ultra-short ctDNA (usctDNA). Here, we developed a non-PCR and non-NGS-based single-droplet-based multiplexing microsensor technology, “Electric-Field-Induced Released and Measurement (EFIRM) Liquid Biopsy” (m-eLB), for lung cancer-associated usctDNA. The m-eLB provides a multiplexable assessment of usctDNA within a single droplet of biofluid in only one well of micro-electrodes, as each electrode is coated with different probes for the ctDNA. This m-eLB prototype demonstrates accuracy for three tyrosine-kinase-inhibitor-related EGFR target sequences in synthetic nucleotides. The accuracy of the multiplexing assay has an area under the curve (AUC) of 0.98 for L858R, 0.94 for Ex19 deletion, and 0.93 for T790M. In combination, the 3 EGFR assay has an AUC of 0.97 for the multiplexing assay.

## 1. Introduction

### 1.1. Liquid Biopsy and Early-Stage Lung Cancer

Non-small-cell lung cancer (NSCLC) is the most common form of lung cancer, accounting for approximately 85% of all cases [1,2,3]. EGFR mutations are found in approximately 10–15% of NSCLC cases [3,4] in the United States and are more commonly found in patients who have never smoked or have a history of light smoking. Traditional methods for detecting EGFR mutations in NSCLC include tissue biopsy, which involves the collection of tissue samples through invasive procedures such as bronchoscopy or surgery. 

Computed tomography (CT) and liquid biopsy are both valuable tools in the early screening and long-term monitoring of lung cancer [5,6]. CT scans are commonly used to screen for lung cancer in high-risk individuals, such as smokers, and can detect early-stage tumors that may not be visible on a chest X-ray. However, the inconvenience of radiation exposure and the false positive rate limit CT for screening for lung cancer. On the other hand, liquid biopsy is a minimally invasive method for detecting genetic mutations and other biomarkers associated with lung cancer, such as circulating tumor DNA (ctDNA) [7,8], circulating tumor cells (CTCs) [9], and protein markers [10]. Liquid biopsy can provide information about tumor heterogeneity and evolution, which can be particularly useful in monitoring disease progression and treatment response over time. In early-stage lung cancer, liquid biopsy has become an increasingly important method for detecting specific mutations in tumor DNA, such as EGFR mutation. Liquid biopsy also has advantages in detecting mutations that may not be present in the primary tumor or metastases, as well as the ability to monitor changes in mutation status over time. 

### 1.2. Ultra-Short Circulating Tumor DNA in Lung Cancer

Ultra-short circulating tumor DNA (usctDNA) is a subtype of circulating tumor DNA (ctDNA) that is characterized by very short fragment lengths, which are typically less than 100 base pairs in length [11,12,13,14]. UsctDNA is thought to be primarily derived from apoptotic and necrotic cancer cells that have undergone further fragmentation. These small DNA fragments are then released into the bloodstream. UsctDNA has been detected in the bloodstream and other body fluids of patients with various types of cancer, including lung cancer [13]. UsctDNA has several potential advantages over longer ctDNA fragments, including increased stability and resistance to nuclease degradation [13].

Recent studies have shown that ultrashort-single-stranded cfDNA (uscfDNA) and usctDNA may have potential clinical utility in the diagnosis and monitoring of lung cancer [13,14]. In addition, biomarker discovery based on uscfDNA with mutations is also important and promising. Many of the biomarker discovery methods used for ctDNA analysis are based on fragmented ctDNA, and there may be a loss or deviation of certain biomarkers when analyzing very short fragments of ctDNA, such as uscfDNA. Developing a biomarker panel specifically for usctDNA could be an important area of research. By focusing on biomarkers that are more readily detectable in usctDNA, researchers may be able to improve the sensitivity and specificity of ctDNA-based diagnostic and prognostic assays for lung cancer.

However, the analysis of usctDNA in lung cancer can be challenging. One of the main challenges in detecting usctDNA is the limitation imposed by the small fragment size of the DNA. The size of usctDNA fragments is typically less than 100 base pairs in length, which makes them difficult to detect using traditional sequencing methods. Detection of mononucleosomal ctDNA routinely involves PCR amplification to locate the rare mutant copies amongst wild-type noise. For PCR-based detection, however, it is difficult to design primers that will reliably amplify the target sequence. This can result in poor sensitivity and specificity and make it challenging to distinguish between usctDNA and non-tumor DNA fragments that may be present in the sample. Some technologies have been developed by adding adapters to the ends of the fragments, which can increase their length and make them easier to detect using standard sequencing methods. This technique is known as adapter-ligation-based amplification (ALA) [15]. However, ALA has the potential for adapter bias, in which some fragments may be preferentially amplified over others, which if applied to usctDNA could lead to a skewed representation in the sample. 

### 1.3. Multiplexing Point-of-Care Device for Lung Cancer Monitor

Point-of-care (POC) devices are medical devices that perform diagnostic tests in close proximity to the patient, with results available quickly and easily [16,17]. In the case of NSCLC and EGFR mutation monitoring, POC devices are particularly useful for the following reasons. Firstly, in early-stage NSCLC, it can be challenging to obtain enough tissue samples for accurate EGFR mutation analysis. Liquid-biopsy-based POC devices can provide an alternative solution by detecting ctDNA in the patient’s blood, allowing for more reliable and timely detection of EGFR mutations [18]. Secondly, patients with NSCLC receiving targeted therapy, such as tyrosine kinase inhibitors (TKIs), require regular monitoring to evaluate treatment efficacy and detect resistance mutations. POC devices that can detect EGFR mutations in blood samples can provide real-time monitoring of the patient’s treatment response and help guide treatment decisions, including the selection of alternative therapies in cases of TKI resistance [19]. In summary, POC devices offer faster turnaround time, reduced cost, and ease of use, which makes them a more practical and convenient option for monitoring EGFR mutations in NSCLC patients.

Historically, liquid biopsy platforms were designed for a limited panel of targets. For example, for NSCLC, EGFR L858R point mutation at exon 21 and deletions within exon 19 were prioritized for their involvement in treatment selection [20]. Recently, further research showed the importance of simultaneous biomarker detection since lung cancer is a complex disease with multiple genetic mutations and alterations that can influence treatment outcomes [21,22]. During early-stage screening for indeterminate pulmonary nodules (IPNs), the tumor burden is typically low, and therefore detection of single cancer-specific biomarkers in blood or other body fluids is challenging and always results in low sensitivity and specificity. Therefore, using a panel of biomarkers that reflect lung cancer in combination with imaging techniques such as low-dose computed tomography (LDCT) may improve early detection rates and reduce the number of false-positive results. This theoretical panel of biomarkers, which includes multiple genetic mutations and protein markers, may provide a comprehensive approach to diagnosis and treatment planning. In addition, in patients undergoing targeted therapy, such as TKI treatment, monitoring a panel of biomarkers that includes genetic mutations associated with TKI resistance, such as T790M [23], C797S [24] in EGFR, MET amplification [25], and HER2 amplification [26], can provide a more comprehensive approach to monitoring treatment response and detecting the emergence of resistance.

### 1.4. Single-Droplet Microarray-Based Multiplexing EFIRM Platform for usctDNA

The requirement of sufficient biomolecule concentration for rare mutation detection in lung cancer liquid biopsy has been a continual challenge. For the main types of liquid biopsy for lung cancer, ctDNA analysis and circulating tumor cell (CTC) analysis require about 10 mL of blood, and exosome analysis typically needs around 5 mL of body fluids. In the case of early-stage lung cancer, the amount of ctDNA or CTCs present in the bloodstream or other body fluids is likely very low, making it more difficult to detect using liquid biopsy. This means that larger sample volumes may be needed to increase the sensitivity of the test. Obtaining even greater sample volumes can be challenging in patients with early-stage disease who may not have large tumor burdens or significant amounts of tumor-derived biomarkers in circulation. In addition, long-term monitoring of lung cancer using liquid biopsy may require repeated sampling over time to detect changes in tumor burden or molecular profile, particularly in response to treatment. Repeated sampling can be difficult for patients, especially if larger sample volumes are required or if invasive procedures such as lung biopsies are needed. This may limit the utility of liquid biopsy for long-term monitoring in some patients.

The electric-field-induced release and measurement (EFIRM) platform is a molecular detection technology that can detect mutations in circulating tumor DNA (ctDNA) from a patient’s blood or saliva sample [27,28,29,30,31]. EFIRM works by applying an electric field to the clinical sample, which induces the release of ctDNA from tumor cells. The released ctDNA is then captured by a microarray, a small chip containing DNA probes that are specific to various genetic mutations. The captured ctDNA is then analyzed using an electrochemical-based method that allows for the detection of specific mutations, such as the EGFR mutation in NSCLC. The EFIRM can detect the presence of mutant ctDNA in a patient’s blood/saliva sample, even at low levels, providing a highly sensitive approach for disease monitoring and the detection of treatment resistance.

Here, we developed a single-droplet multiplex EFIRM liquid biopsy (m-eLB) platform that can directly detect ultra-short DNA fragments. The original EFIRM demonstrated the ability to directly detect usctDNA without PCR amplification [14,32]. The shortest fragment length that EFIRM can detect is around 30 bp of oligonucleotide without the use of adapters or ligands. EFIRM can uniquely provide a direct measurement of usctDNA without DNA extraction. In this proof-of-concept study, we show the m-eLB can simultaneously detect multiple usctDNA synthetic oligos in a single drop of biofluid, allowing for a more comprehensive analysis of the patient’s disease status. This can provide a more complete picture of the patient’s disease and can be used for early detection, disease monitoring, and treatment guidance.

## 2. Results

### 2.1. m-eLB Sensor for Multiple EGFR Mutations

**Figure 1:**
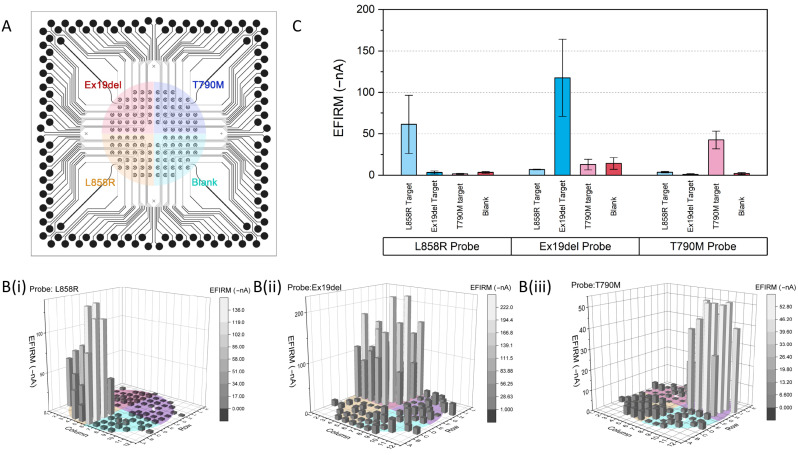
Specificity of three EGFR mutation targets on EFIRM microsensor. (**A**) Distribution of three EGFR targets and blank control on the sensors. EFIRM reading with sensors coated with single EGFR probe for (**B**(**i**))-L858R; (**B**(**ii**))-Ex19del; (**B**(**iii**))-T790M for each individual electrode. Color maps are provided to indicate the EFIRM current reading. (**C**) Averaged amperometric signal reading from L858R/Ex19del/T790M-coated sensor from each group on targets; two standard deviations are included in the error bar.

The EFIRM sensors were first precoated with a single EGFR probe and assayed for multiple targets. Three EGFR probes—L858R, Ex19del, and T790M—were electrochemically polymerized onto the sensor by a single droplet of coating buffer. According to the specificity and sensitivity of m-eLB (Supplementary Materials Figures S1 and S2), each sensor chip was then assayed for the three EGFR mutation targets at 100 pM, as well as a blank control (hybridization buffer only). The location of the three targets and blank is in the four quadrants of the 96-array; four different droplets of target were dropped onto the surface, as illustrated in Figure 1A. Each droplet was around 100 μL. Individual readings of each electrode are shown in Figure 1B, and the averaged data for each target are shown in Figure 1C. For each EGFR mutation probe, the sensor only shows a positive signal for its correspondent target, whereas a negative signal is given for other EGFR targets, including the blank. The signal-to-noise ratio (SNR) of the L858R probe was 18.0 for Ex19del, 35.7 for T790M, and 17.8 for the blank. The SNR of the Ex19del probe was 17.0 for L858R, 9.1 for T790M, and 8.3 for the blank. The SNR of the T790M probe was 11.6 for L858R, 46.1 for Ex19del, and 20.9 for the blank. The total reaction time was around 45 min. Signal difference was observed within each quadrant, possibly due to the different distributions of target in the droplets on the surface. 

### 2.2. Multiplexing DNA Measurement for Three EGFR Mutations in a Single Droplet

**Figure 2:**
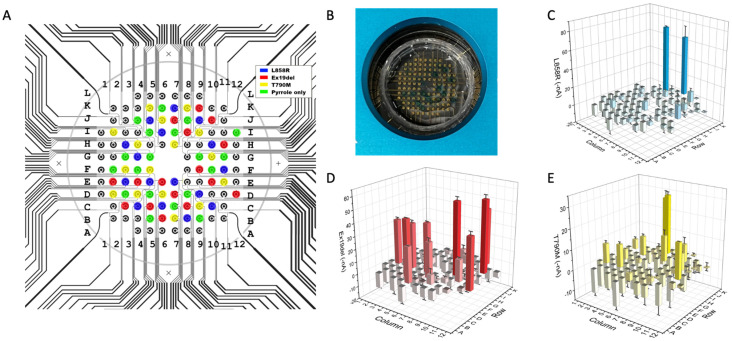
Multiplexing EFIRM sensor for EGFR mutation detection. (**A**) Overlay of pattern for microprinting EGFR probes and the circuit design with three EGFR sequences and pyrrole-only control. (**B**) Picture of amperometric reading with TMB substrate. Blue dots indicate positive signal. (**C**) EFIRM reading of L858R targets. (**D**) Ex19del targets. (**E**) T790M targets. Blank electrodes were disconnected as location markers. Bar charts are based on duplicated assay with standard deviation shown as error bars.

For a single-droplet-based multiplexing assay, the context of use is that for each individual clinical sample, multiple biomarkers need to be assayed simultaneously in one reaction well. Therefore, multiple probes for a biomarker panel need to be pre-immobilized onto the sensor at pre-designated locations for the same sample droplet. Since the EFIRM probe immobilization is a rapid aqueous-based electrochemical polymerization for only 8 s, a microprinting platform is combined with the EFIRM platform to undertake rapid fabrication and measurement in situ. The total time taken for printing three EGFR probes and a blank control is 5 min or less, followed by the 8 s polymerization (Figure 2A). This is followed by the EFIRM sample measurement using our homemade connection to the multichannel potentiostat. Amperometric currents for all 96 channels are read out simultaneously. 

Since the amperometric reading is based on a TMB substrate with a couple redox procedure between HRP, hydrogen peroxide, and the TMB mediator, the TMB will turn blue for a positive reading (Figure 2B). However, the colorimetric reaction is not localized, and it is not accurate to measure the color, especially for high-throughput microarrays. In addition, colorimetric measurement requires additional optical instrumentation, including a light source, optical pathway alignment, and optical grating to measure specific wavelengths to avoid non-specific signals. Here, the EFIRM directly measures the redox current, utilizing the same instrumentation for polymerization and hybridization. The 2-D mapping of the current in –nA is shown in Figure 2C–E after background subtraction. A high reading in the negative current indicates a positive signal. A low current suggests a negative signal.

### 2.3. Acurracy of m-eLB Platform for usctDNA

**Figure 3:**
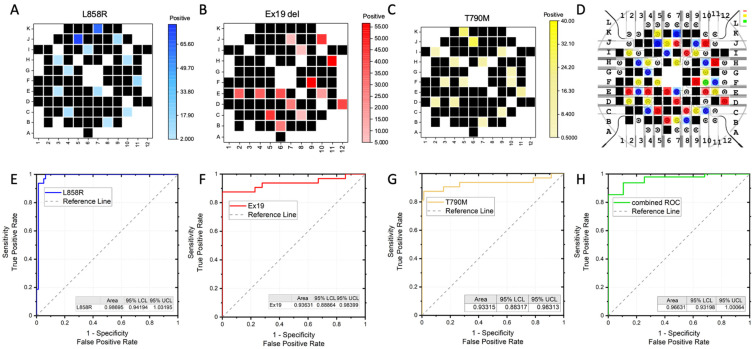
Accuracy of EFIRM multiplexing microsensors with EFIRM reading for (**A**) L858R, (**B**) Ex19del, (**C**) T790M, and (**D**) overlay of 3 EGFR mutation EFIRM results with designed distribution of EGFR markers. The accuracy of the EFIRM sensor is analyzed by receiver operating characteristics for (**E**) L858R, AUC = 0.98; (**F**) Ex19del, AUC = 0.94; (**G**) T790M, AUC = 0.93; and (**H**) combined 3 EGFR, AUC = 0.97. The 95% confidence interval of the AUC is listed as the lower confidence limit (LCL) and upper confidence limit (UCL) on each plot.

The accuracy of the EFIRM microsensor has been illustrated in Figure 3A–D. The accumulated EFIRM results from the 3 EGFR sequence are overlayed with the original design of the microprinted pattern. It shows that all the EGFR-positive probes detected a positive EFIRM signal and that all the negative electrodes only gave a low EFIRM reading. For quantitative analysis, receiver operator characteristics (ROC) were obtained. For each EGFR mutation probe, the individual ROC curves are shown in Figure 3E–G, with the area under the curve (AUC) of L858R being 0.98, that of Ex19del being 0.94, and that of T790M being 0.93. The combinational ROC for the three markers is also provided (Figure 3H) and has an AUC of 0.97. Those results suggest the high sensitivity and specificity of the EFIRM microsensor for EGFR mutation. 

The results demonstrate that the m-eLB platform is capable of performing multiplex biomarker detection from a single sample droplet. Although this is a proof-of-concept prototype with oligonucleotides in a model system, it shows the novelty of a single-droplet liquid biopsy assay in one reaction for the detection of multiple lung cancer markers. With a limited sample volume (~400 µL) and a miniaturized size of the sensor (total area around 1 cm^2^), there are notable challenges for both sensitivity and specificity (including cross-reaction between different sensors in the simultaneous assay). The total reaction time takes place within 1 h. Our recent study shows that introducing chaotropic ions into both the sample collection and assay will significantly improve the sensitivity of eLB to low copy numbers of usctDNA directly in a sample of less than 10 µL. The current sample volume used in this study is around 400 µL, in order to cover the whole sensor chip and form a stable liquid environment. Regarding the possible throughput of the m-eLB (up to 96 assays in one well), less than 5 µL is used for each electrode. In addition, eLB has already shown multiplexibility for multiple types of targets in previous studies, including the measurement of protein, DNA, and RNA simultaneously on the same sensor chip [33,34]. According to the COSMIC database, a biomarker panel with 14-plex-circulating lung tumor ctDNA and a 6-plex miRNA biomarker panel can provide 85% coverage for early-stage lung cancer assessment. Our further work will develop the m-eLB into a 14-plex ctDNA and 6-plex miRNA LCBP, truly multiplexing. 

In our previous study, eLB showed specificity in differentiating down to a 0.1% target with a large amount of other interferents [18]. This proof-of-concept study also suggests very limited cross-reactivity between different markers (AUC > 0.9 for all three assays). Although m-eLB shows good differentiation between different usctDNA sequences, in the context of clinical sample measurement, the interferents for m-eLB are not limited to different ctDNA sequences only. The complexity of the biomatrix from body fluids is presented in the mixture of cells and a multitude of molecules dissolved within it, such as proteins, electrolytes, hormones, enzymes, metabolites, and waste products. Specifically for saliva, the high viscosity from mucin will also cause interference with the eLB assay. According to the previous eLB application in plasma and saliva, those interferents did not cause a significant signal in the eLB assay [18,19,30,33]. To further eliminate the biomatrix effect on m-eLB, sample pre-treatment will be applied for additional quality security. In the saliva usctDNA assay, a sample collector has been developed to remove the majority junk of the matrix that may interfere with the assay, including cells, while still preserving the protein and nucleotide in the samples [35].

The results indicate there is potential for direct measurement of ultra-short oligonucleotides. Previous literature suggests that ultra-short ctDNA (~40 bp) (usctDNA) is present in the plasma and saliva of NSCLC patients [13,14,32]. In limited samples, usctDNA was detected by targeted sequencing. Those reports indicate that usctDNA is a novel type of candidate for liquid biopsy. In our previous study on usctDNA in lung cancer plasma and saliva, comparisons between EFIRM and ddPCR were carried out side by side [14]. In a plasma sample from NSCLC, ddPCR can detect a portion of the positive subjects, whereas EFIRM has around 100% sensitivity. In saliva samples, ddPCR can rarely detect the positive subject, whereas EFIRM can still detect all the positive samples. This preliminary study indicates that EFIRM is very efficient in its ability to directly detect usctDNA and mncfDNA, whereas PCR-based technologies cannot. The theoretical dynamic range of EFIRM for usctDNA is from 40 bp to 160 bp [14]. 

## 3. Materials and Methods

### 3.1. m-eLB Sensor Fabrication

**Figure 4:**
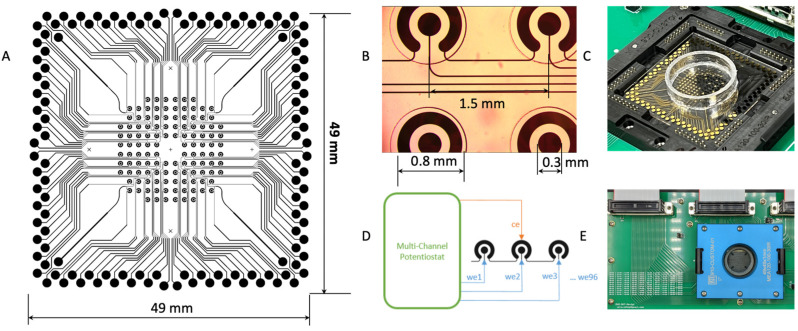
Multiplexing EFIRM biosensor for lung cancer. (**A**) Circuit design of the 96-array microsensor. (**B**) Structure of each individual 2-electrode electrochemical sensor. (**C**) Microprinted EFIRM sensor with mounted well. (**D**) Electrical connection of the array of electrodes. (**E**) Adapter for sensor connection to multichannel potentiostat.

The microchip was fabricated with a 100 nm gold film deposited on a glass substrate using E-beam evaporation for a smooth surface (Platypus Technologies, Madison, WI, USA). The microchip consists of 96 individual electrochemical cells, and the cells are isolated with an epoxy-based negative photoresist SU-8 layer on top of the final chip (Figure 4A,B). The microchip uses a two-electrode system (working/counter) with the counter electrode (CE) shared on the potentiostat for multiplexing. Each cell has a diameter of 2 mm, and they are placed 1.5 mm apart (Figure 4B). With a BioDOT multichannel microprinter (Biodot OmniaTM, Irvine, CA, USA), around 50 pL of each probe was precisely printed onto each electrode at a specified location (Figure 4C). For the multiplexing assay, multiple probes were printed onto different locations, together with negative controls. To avoid evaporation during printing, humidity is strictly controlled.

A custom adapter was designed to interface the microchip with a multichannel potentiostat (Figure 4D,E) (IVIUM CompactStat.h with 3 multiWE32, Eindhoven, The Netherlands). The potentiostat can control and measure the 96 electrodes simultaneously.

### 3.2. m-eLB Platform with Multi-Channel Potentiostat

The basic EFIRM assay has been previously described in detail [18,33,36]. The single-droplet EFIRM multiplexing assay utilizes a 150 μL clinical sample without any pretreatment. Paired probes (capture and detector; Integrated DNA Technologies, San Diego, CA, USA) specific for the three TKI EGFR mutations were designed for EFIRM as shown in Table 1. The targeted sequences for all three are around 50 bp in length.

**Figure 5:**
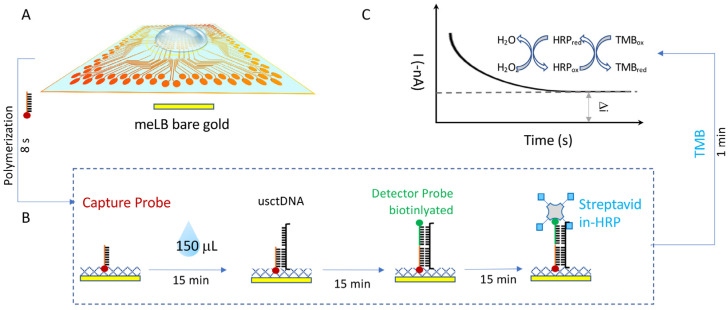
Illustration of high-throughput multiplexing EFIRM assay. (**A**) Microarray for single droplet-based lung cancer liquid biopsy. (**B**) Procedure of usctDNA EFIRM assay. (**C**) Amperometric reaction for EFIRM assay between HRP label and TMB substrate.

The whole procedure is illustrated in Figure 5. All the capture probes were unlabeled. All the detector probes were biotinylated on the 3′ end. The capture probes (100 nmol/L) were first co-polymerized with pyrrole onto the bare gold electrodes by applying a cyclic square-wave electric field at 300 mV for 1 s and 1100 mV for 1 s for four cycles. The sensor was then washed with 1× PBST buffer (Thermo Fisher, Waltham, MA, USA). Hybridization was performed in 300 μL of ultra-sensitive hybridization buffer (Thermo Fisher Scientific, Waltham, MA, USA), spiked with a single oligonucleotide or a mixture of more than one. The EFIRM condition was 300 mV for 1 s and 500 mV for 1 s for a total of 150 cycles of 2 s, each followed by a 15-minute incubation at room temperature. After washing, the detector probes were mixed with casein-phosphate buffered saline (Invitrogen, Carlsbad, CA, USA) at a 1:100 dilution and transferred onto the electrodes. Subsequently, streptavidin poly-HRP80 conjugate (Fitzgerald Industries, Acton, MA, USA) was mixed with casein-phosphate-buffered saline (Invitrogen) at a 1:3 ratio and incubated for 15 min. Amperometric current was finally measured in TMB (3,3′,5,5′-tetramethylbenzidine) at −200 mV for 1 min.

## 4. Conclusions

The m-eLB enables the simultaneous detection of multiple mutations in a single droplet of sample, making it a comprehensive and efficient diagnostic tool for lung cancer monitoring. The eventual direct measurement of multiple usctDNA in a clinical sample is not only critical for ctDNA diagnosis but also for the discovery of new usctDNA biomarkers correlated to disease conditions. Therefore, in the future, the EFIRM platform’s electrochemical detection method combined with microarray technology can offer significant benefits for EGFR mutation monitoring in lung cancer patients. 

## Figures and Tables

**Table 1:** EFIRM probes for three tyrosine kinase inhibitor mutations in EGFR for lung cancer.

EGFR Mutation	Oligo Type	Sequence (5′-3′)
L858R	Capture probe	AAAAAAAAAAGAAATAAACAAATAAAACAATAACAAATAAAAAAAAACAAATAAACAATAAAAAAAAACAA GTTTGACCCGCCCA
	Detector probe	AAAATCTGTGATCTTGACATGCTGCGGTGTTTTCACCAG-/biotin/
	Ultra-short target	CTGGTGAAAACACCGCAGCATGTCAAGATCACAGATTTTGGGCGGGCCAAACTG (54 bp)
Ex19 deletion	Capture probe	AAAAAAAAAAAATAAAAAAAAAAAAATAAAAAAAAAAAAATAAAAAAAAAAAAATAAAAAAAAACGCTTTCGGAGATGTTTTGATAGC
	Detector probe	GACGGGAATTTTAACTTTCTCACCTTC-/biotin/
	Ultra-short target	GAAGGTGAGAAAGTTAAAATTCCCGTCGCTATCAAAACATCTCCGAAAGC (50 bp)
T790M	Capture probe	AAAAAAAAAAGAAATAAACAAATAAAACAATAACAAATAAAAAAAAACAAATAAACAATAAAAAAAAACAA GAGCGGCATGATGA
	Detector probe	GCTGCACGGTGGAGGTGAGGCAGATGCCCAGC-/biotin/
	Ultra-short target	GCTGGGCATCTGCCTCACCTCCACCGTGCAGCTCATCATGCAGCTCATGCCC (52 bp)

## Data Availability

All data are available from authors upon request.

## Supplementary Materials

The following supporting information can be downloaded at: https://www.mdpi.com/article/10.3390/ijms241210387/s1, [37].
