# Supplementary material for: Single-Droplet Microsensor for Ultra-Short Circulating EFGR Mutation Detection in Lung Cancer Based on Multiplex EFIRM Liquid Biopsy
Source: Int J Mol Sci. 2023 Jun 20;24(12):10387. doi: 10.3390/ijms241210387 (PMC10299723; doi:10.3390/ijms241210387)
Supplement: Supplementary file 1 [file ijms-24-10387-s001.zip › ijms-2408221-supplementary.pdf]

Supplementary Materials

Single Droplet Microsensor for ultra-short circulating EFGR mutations detection in Lung Cancer based on Multiplex EFIRM Liquid Biopsy

m-eLB capture probe polymerization

The m-eLB capture probe is immobilized onto the surface by copolymerization of oligo capture probe with pyrrole. The detailed protocol has been described in our previous publication. In brief, 1.25 uM of capture probe and 1:200 diluted pyrrole was well mixed in 0.3 M KCl. Then droplet of the mixture is placed onto the electrode. A cyclic square wave with 4 cycles of 300 mV and 1100 mV is applied. Total time is 8 seconds. The surface characterization is discussed in the previous publication[1].

m-eLB amperometric assay

The final m-eLB reading is based on amperometric assay between HRP and TMB (3,3',5,5'-Tetramethylbenzidine) . The electrochemical current is reading at -200 mV for 60 seconds. Reported signal is the average of last 5 seconds of current presented in -nA. The amperometric readouts are shown in Figure S1 for different probes.

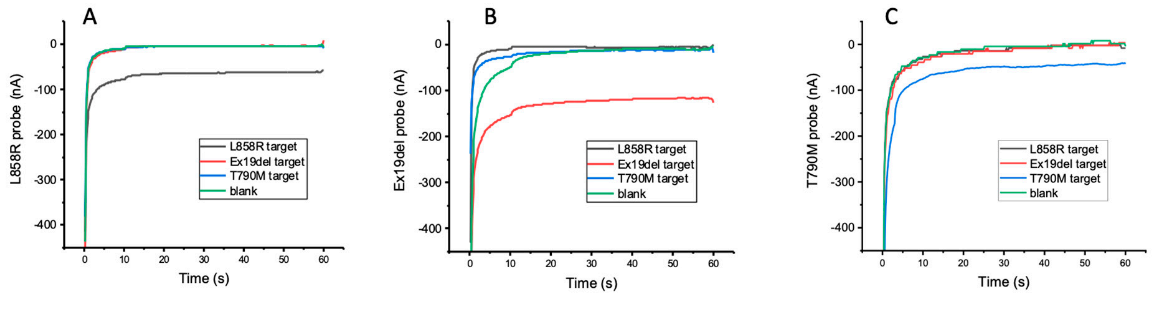

Figure S1. Specificity of the m-eLB microarray for three EGFR mutation detection for (A) L858R (B) Ex19del and (C) T790M oligonucleotide. The curves are amperometric reading from microelectrodes.

m-eLB sensitivity and specificity

The sensitivity of m-eLB is studied with oligos in buffer for this proof-of-concept study. Droplets of serials titrated EGFR oligo in hybridization buffer is placed on different location of the same sensor chip in the sample hybridization step. The signals of each concentration is presented by average of all the electrodes covered with the droplets. There are around 5-10 electrodes covered by the individual droplets. The standard curves are listed in Figure S2 for L858R, Ex19del and T790M. Due to the small size of the micro-electrode, the current sensitivity of the m-eLB is at around 10-1 pM. For the following experiments, concentration of 100 pM was chosen to get high enough signal for assays.

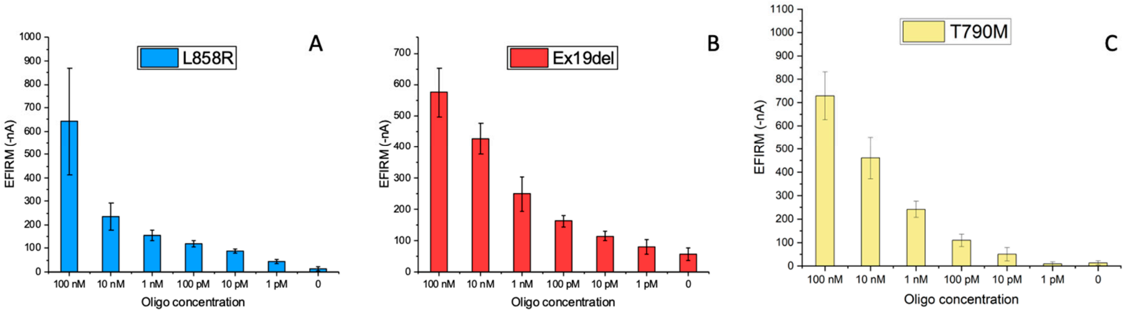

Figure S2. Sensitivity of the m-eLB microarray for three EGFR mutation detection for (A) L858R (B) Ex19del and (C) T790M oligonucleotide. The concentration is from 100 nM to 1 pM of oligos.

The specificity of m-eLB is investigated by the quadrant assay described in the main text. Three EGFR probes included L858R, Ex19del and T790M were electrochemically polymerized onto the sensor by single droplet of coating buffer. Then each sensor chip was assayed for the three EGFR mutation targets at 100 pM, as well as a blank control (hybridization buffer only). The location of the 3 targets and blank is at the four quadrants of the 96-array by drop 4 different droplets of target onto the surface as illustrated in Figure 1A. The amperometric reading along with time are shown in Figure S1, corresponding to the bar chart in Figure 1C.

1. Wei F, Liao W, Xu Z, Yang Y, Wong DT, Ho CM. Bio/abiotic interface constructed from nanoscale DNA dendrimer and conducting polymer for ultrasensitive biomolecular diagnosis. Small. 2009;5(15):1784-90.
